# Design and Construction of a Focused DNA-Encoded Library for Multivalent Chromatin Reader Proteins

**DOI:** 10.3390/molecules25040979

**Published:** 2020-02-22

**Authors:** Justin M. Rectenwald, Shiva Krishna Reddy Guduru, Zhao Dang, Leonard B. Collins, Yi-En Liao, Jacqueline L. Norris-Drouin, Stephanie H. Cholensky, Kyle W. Kaufmann, Scott M. Hammond, Dmitri B. Kireev, Stephen V. Frye, Kenneth H. Pearce

**Affiliations:** 1 UNC School of Medicine, Department of Biochemistry and Biophysics, University of North Carolina at Chapel Hill, Chapel Hill, NC 27599, USA; jmr5808@email.unc.edu; 2 UNC Eshelman School of Pharmacy, Division of Chemical Biology and Medicinal Chemistry, Center for Integrative Chemical Biology and Drug Discovery, University of North Carolina at Chapel Hill, Chapel Hill, NC 27599, USA; shivag@email.unc.edu (S.K.R.G.); dangzhaoo@gmail.com (Z.D.); yienliao@live.unc.edu (Y.-E.L.); jnorris@unc.edu (J.L.N.-D.); cholensk@email.unc.edu (S.H.C.); dmitri.kireev@unc.edu (D.B.K.); 3 UNC Gillings School of Public Health, Biomarker Mass Spectrometry Facility, University of North Carolina at Chapel Hill, Chapel Hill, NC 27599, USA; lbcollin@email.unc.edu; 4 UNC School of Medicine, Department of Cell Biology and Physiology, University of North Carolina at Chapel Hill, Chapel Hill, NC 27599, USA; kyle_kaufmann@med.unc.edu (K.W.K.); scott_hammond@med.unc.edu (S.M.H.)

**Keywords:** DNA-Encoded Libraries, target-class drug discovery, chromatin reader protein

## Abstract

Chromatin structure and function, and consequently cellular phenotype, is regulated in part by a network of chromatin-modifying enzymes that place post-translational modifications (PTMs) on histone tails. These marks serve as recruitment sites for other chromatin regulatory complexes that ‘read’ these PTMs. High-quality chemical probes that can block reader functions of proteins involved in chromatin regulation are important tools to improve our understanding of pathways involved in chromatin dynamics. Insight into the intricate system of chromatin PTMs and their context within the epigenome is also therapeutically important as misregulation of this complex system is implicated in numerous human diseases. Using computational methods, along with structure-based knowledge, we have designed and constructed a focused DNA-Encoded Library (DEL) containing approximately 60,000 compounds targeting bi-valent methyl-lysine (Kme) reader domains. Additionally, we have constructed DNA-barcoded control compounds to allow optimization of selection conditions using a model Kme reader domain. We anticipate that this target-class focused approach will serve as a new method for rapid discovery of inhibitors for multivalent chromatin reader domains.

## 1. Introduction

One mechanism for the modulation of chromatin structure and dynamics is through post-translational modifications (PTMs) on the N-terminal tails of histone proteins found in nucleosomes. The biological consequences associated with PTMs result from recruitment of regulatory complexes through “reader” domains that recognize them, either individually, or in combination. Having multiple molecular recognition events, e.g., binding to multiple PTMs, occurring simultaneously deems the interaction multivalent [1,2]. Combinatorial recognition of PTMs facilitates a flexible and dynamic system for controlling important biological processes by creating an additional level of specificity over a singular PTM recognition mechanism. An increasing number of proteins having multivalent interactions with PTMs are being revealed regularly with many of these proteins containing at least two adjacent methyl-lysine (Kme) reader domains [3,4,5,6,7,8,9]. Furthermore, these multivalent proteins are of therapeutic interest due to their association with numerous human diseases (Table 1).

Histone lysine residues mono-, di-, or trimethylated at the *ε*-amino position are some of the most common chromatin PTMs. The Kme reader proteins that recognize these modifications can be grouped into four general families identified as Ankyrin repeats, WD-40 repeat domains, plant homeodomain (PHD) fingers, and Royal family proteins [10]. A general characteristic of Kme readers is the formation of a binding pocket cage made of 2–4 aromatic residues, which coordinates the modified residue [11]. A cation-π interaction drives the interaction, but hydrophobic and van der Waals contacts can also play a role in binding the residue [12]. Selectivity of Kme reader proteins for these PTMs can be achieved by differences in methylation state and the surrounding sequence of the lysine residue, for instance, the correct position on the histone tail. Additionally, neighboring PTMs to the primary modification can also enhance or inhibit binding to the histone tail [2].

Several features of the Kme reader proteins make this an attractive target class family for small molecule and/or peptidomimetic inhibitor discovery [10,11,13,14]: 1) there are over 200 Kme readers present in the human proteome; 2) each domain contains a shared common binding motif; 3) structural insight exists on how to potentially gain selectivity; and 4) misregulation of Kme proteins is associated with several diseases. Target class drug discovery is a systematic strategy that utilizes established knowledge in chemistry, biology, and structural biology to leverage and rationalize plans for discovering new, selective inhibitors. There are several key pillars for target class drug discovery: a structural-knowledge foundation, focused compound libraries, and an assay platform that can support robust structure-activity relationship (SAR) development [13]. Structural knowledge, perhaps from a crystal structure with a native ligand or hit compound, can inform decisions on designing new compounds to confer greater potency and selectivity. This information can also inform the design and assembly of a focused compound library, expanding from the original ligand or hit compound, into broader chemical space. When tied together with a screening platform that is amenable to several representative members in a protein family, SAR profiles across a target class can be achieved.

An initial hit molecule is typically required to initiate a target class drug discovery campaign. Traditional high-throughput screening (HTS), which involves amassing large libraries of individually stored compounds and then testing them against a target of interest through bioassays, has been a reliable strategy for hit compound discovery. Impressively, throughout both academic and industrial labs, the size of traditional chemical screening libraries is typically in the tens of thousands to several millions of compounds. Although this approach for HTS is usually automated, it is expensive to produce, maintain, and screen a library [15,16]. Over the past decade or so, DNA-Encoded Library (DEL) technology has revolutionized the practice of HTS. Importantly, and predominately within industry, encoded libraries can exceed billions of compounds to cover a vast amount of chemical space and the screening methods are more rapid and much less expensive compared to traditional HTS [17].

Large libraries that contain random chemical diversity can increase chances for success for even traditionally difficult targets generally deemed ‘undruggable’. However, it has also become apparent that library size does not necessarily correlate to hit rate [18,19,20]. Much like focused screens in traditional HTS, for example kinase-focused sets that retain the ‘hinge-binding motif’ [21,22], using structure-based information and computational methods for DEL design can improve success rates using smaller libraries (thousands to millions) [18,23]. This target class approach is advantageous in that less resources are required to make smaller libraries essentially pre-enriched for compounds with specialized features for the target class [24]. Moreover, focused DELs can be readily designed by ligand- and structure-based computational tools. Both library design and screening data analysis can be computationally enhanced to maximize the success rate. For instance, due to structural homology between the family member proteins, fragments appearing in ligands of any family member might fit structural microenvironments in multiple other members. It also allows for easier processing of data and hit validation. Because of the relative increase in size of DELs compared to traditional libraries, this form of HTS can, even in the context of a target-class focus, also generate unanticipated ligands and binding modes.

Here, we have utilized structural and fragment-based information to design and produce a targeted library containing features known to be important for binding Kme reader domains, especially ones that contain multiple binding sites. We also provide experimental evidence of selection conditions and data supporting the ability to successfully select for a positive control compound within a DEL using a model Kme reader system.

## 2. Results and Discussion

**Figure 1:**
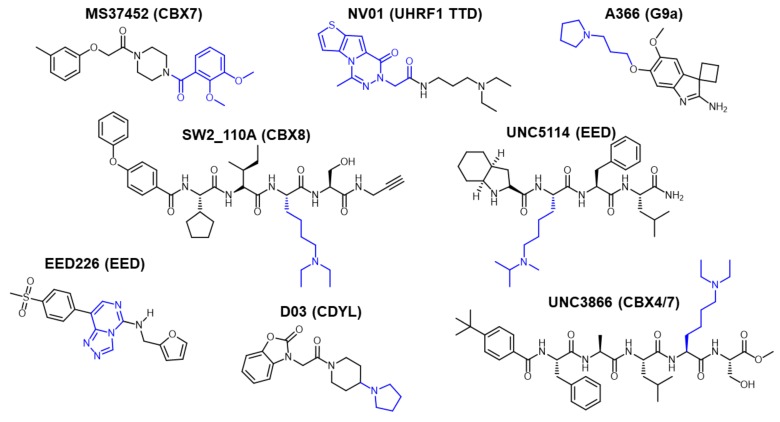
Examples of methyl-lysine domain inhibitors with the functionality that interacts with the methyl-lysine pocket highlighted in blue. Protein targets are included in paratheses.

Over the past few years, several labs, including ours, have published small molecule inhibitors of Kme readers [53,54,55,56,57,58,59,60,61] (Figure 1). We have been utilizing a target class platform for Kme reader proteins and have gained insight into the types of molecules that bind to Kme readers [53,56,62,63,64]. To continue advancing our capabilities, we chose to begin developing a platform of focused DELs for Kme reader proteins. To tackle this, we combined computational studies and structure-based knowledge of Kme reader hits and probes to design and produce several, small, focused DELs. In addition to producing our focused libraries, we completed several experiments using a model Kme reader system to validate DEL selection conditions.

### 2.1. Computational Selection of the Library Building Blocks

**Figure 2:**
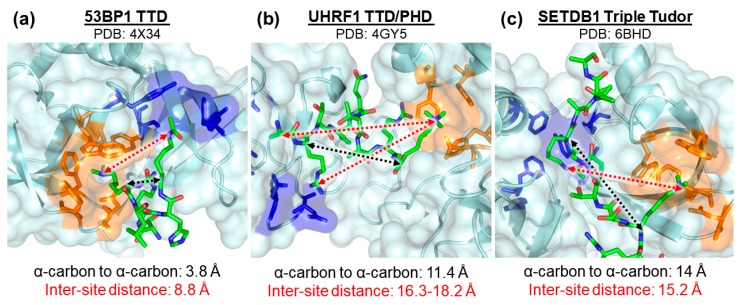
Close-up view showing basic dimensions and spatial features for multivalent Kme reader: peptide interactions of proteins (**a**) 53BP1 (PDB: 4X34), (**b**) UHRF1 (PDB: 4GY5), and (**c**) SETDB1 (PDB: 6BHD). The regions highlighted in orange of each panel are the methyl-lysine binding pockets and the regions highlighted in blue are the ancillary binding pockets. The distances between the α-carbon of each PTM residue and the inter-site distances are depicted using black and red arrows, respectively.

The rationale behind our library was to exploit the general structural architecture of the Kme reader proteins (Figure 2). The library compounds are intended to be bivalent binders consisting of (i) a Kme-cage-binding fragment, (ii) a fragment binding either a hydrophobic patch or a small pocket, such as another Kme or acetyl-lysine (KAc) binding site, nearby the cage, and (iii) a linker between (i) and (ii). Our in-house library was used to sample a diverse set of Kme-mimic fragments. All of them feature a secondary, tertiary or quaternary amine as proxies to mono-, di- or trimethylated lysine side chains. A total of 1,337 fragment-like lysine analogs resulting from our previous projects [53,54,56,62] were found in the internal database. They were clustered using k-means algorithm in the functional circular fingerprints (FCFP) descriptor space [62] with a Tanimoto similarity inclusion criterion of 0.5. A total of 171 cluster centers were retained as potential library components. Fragments for an adjacent site were selected to maximize property-based diversity. All available fragment-like compounds in the internal database were clustered using whole-molecule descriptors, such as, octanol-water partition coefficient (logP), Solvent Accessible Surface Area (SASA), counts of hydrogen bond donors and acceptors, as well as a number of shape and size descriptors. A total of 128 fragments were selected to construct bivalent library compounds. Finally, the linker length was evaluated using an ad hoc structural analysis. Several diverse reader proteins (53BP1, EED, UHRF1) were selected for a docking study along with a few hundred computationally enumerated library compounds. The ligands were constrained to bind to the Kme-binding pocket by its Kme-mimic portion. We then verified whether the linker allows the second fragment to meaningfully interact with the protein by forming a network of polar and/or non-polar interactions. The selected fragments were then also used to enumerate the whole library and provide structures during the post-screening analyses.

### 2.2. Summary of DEL Production Strategy

**Figure 3:**
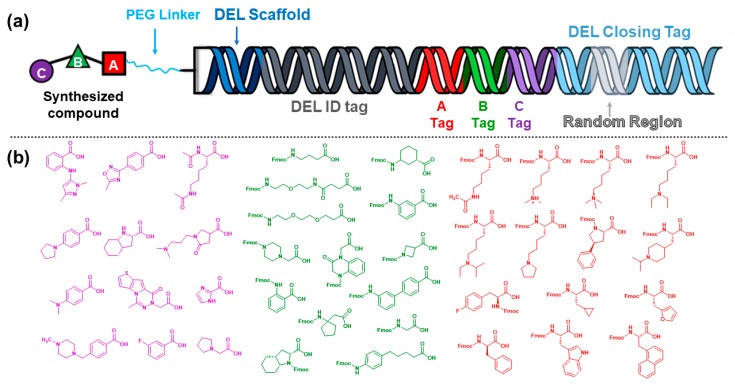
(**a**) A single barcode representation of a library member with important components identified. (**b**) Examples of building blocks utilized in the library described (UNCDEL003).

The approach for library construction that we chose is a linear fragment assembly with split and pool synthesis for chemical couplings and DNA barcode ligations (Figure 3A). Our DNA scaffold contains two short complementary single-stranded oligonucleotides coupled covalently with flexible spacers similar to that reported by Clark et al. [65,66]. Following ligation of the initial primer, an Fmoc-protected PEG linker was coupled to the scaffold and serves as a spacer between the DNA sequence and the library compound. Following Fmoc deprotection, the free amine on the spacer acted as the starting point for the synthesis of library members from the C to N termini. Our DEL members were coupled in three cycles. We chose Fmoc-protected compounds, or building blocks, for the first two cycles of synthesis and carboxylic acids for the third cycle (Figure 3B, Supplemental Material). An EDC/HOAt/DIPEA coupling combination was chosen for amide bond formation as a previous investigation demonstrated high yields for most building blocks tested [67]. The building blocks utilized in our libraries were tested for the ability to couple to the PEG linker that was attached to the DNA scaffold-initial primer conjugate with >80% yield of the expected product. Lysine mimetics were incorporated as building blocks in cycles 1 and 3, and some were incorporated in cycle 2 as linker moieties.

When considering our targets of interest, we took into account that many of the proteins achieve selectivity through surface grooves with which the histone tail ligands interact. These grooves are adjacent to the pockets that bind the methylated lysine residues. Often, Kme readers only interact with a small portion of the histone tail, with entry and exits points of interaction. This feature enables access to the Kme pocket in a bi-directional manner, doubling the potential orientation in which our molecules can potentially bind the protein of interest. Many of the second cycle building blocks were incorporated into our library as linkers that could lie within these surface grooves and also physically link two Kme mimetics with the capability to reach adjacent pockets (Figure 3B).

To record the small molecule library members being produced, the end of the library scaffold contains a three-base 3′ overhang, providing a mechanism for subsequent DNA tag ligation. A double-stranded oligonucleotide is ligated to the end of the scaffold and acts as the primary DEL identifier. This primary library tag contains a two-base 3′ overhang suitable for the proceeding tag. The DNA tags that serve as codes for building block addition are short, unique double-stranded DNA sequences. Each tag has an eight-base variable region and a two-base 3′ overhang. The overhang of each tag is designed for a specific step during library synthesis so only new tags can ligate to the DNA constructs from the preceding cycle; not to a truncated barcode. Following the third cycle of library production, a final closing primer tag is ligated to the end of all the sequences. This tag contains a region of seven randomized nucleotides that provides unique molecular identifiers (UMI) for library members, important for reducing PCR and sequencing biases (Figure S7).

### 2.3. Production of Barcoded Control Compounds to Inform DEL Selection Conditions

Targeting chromatin reader proteins using DELs requires an approach to diminish non-specific DNA interactions interfering during the selections, as many of these proteins are highly basic and in fact coordinate with DNA [68,69,70]. Studies from the Krusemark lab have shown the utility of DELs for chromodomain containing proteins by producing a DEL of peptidomimetics [71,72]. To address the feasibility of DELs for chromatin readers in our hands and to establish general selection and sequencing protocols, we explored a model system using the chromodomain-containing Kme reader protein CBX7, which is part of the multi-subunit protein complex Polycomb Repressive Complex 1 [56]. We selected CBX7 as a model reader protein, since it is known to interact with nucleotides [68], has a well-defined chemical probe UNC3866, and has a negative control UNC4219. To do so, we produced barcoded versions of the positive and negative control probes with unique DNA sequences (Figure S1A). The unique barcodes allow for selective amplification of the compounds by an associated PCR primer set even when combined. First, we ensured that both barcodes would amplify with the correct primer sets selectively (Figure S1B).

With selective amplification enabled, we then tested for optimal conditions that could be used for DEL selections. Using magnetic Dynabeads as our immobilization strategy, we completed selection experiments with varying buffers, salt concentration, incubation times, and washes and analyzed differences in positive and negative control retention using quantitative PCR (qPCR). We completed the investigation of conditions using a 6X-his-tagged CBX7 (CBX7-his) and biotinylated CBX7 (CBX7-avi) to make comparisons of tag influence. After several experiments we were able to successfully retain the positive control versus the negative control. Examples of some of those experiments are summarized in Figure S1C,D. In general, trends suggest increasing the concentration of salt increases the difference in cycle at which each compound’s barcode amplifies. Additionally, using the biotinylated CBX7 generates a larger cycle difference versus the his-tag protein. Using this qPCR approach, we determined the following as final, optimized conditions for CBX7: Buffer – 20 mM Tris-HCl, pH 7.5, 300 mM NaCl, 0.05% Tween-20, and 2 mM DTT, 1 µM biotinylated protein, three rounds of a one hour incubation followed by 1 wash and heat elution of 10 min at 80 °C. These results with DNA barcoded control compounds binding a Kme reader with known nucleotide binding activity laid the foundation for optimal conditions for conducting DEL selections.

### 2.4. Evidence of Selection Power Using Control Compounds

After determining selection conditions, we produced two barcoded control compounds that contain the same library associated tags which allow them to be incorporated into one of our libraries, UNCDEL003 (Supplemental). These barcoded compounds were incorporated into two separate final library preps, or those that had the closing primer ligated at separate times, for completing selections in duplicate. The barcoded controls were integrated at a concentration representative of a single DEL member, with the accuracy of our concentrations confirmed by qPCR (Figure S1E). We then completed selections using our UNCDEL003 preparations that contained the controls. The selections involved incubating bead-immobilized CBX7 with the DEL for 1 h, removing the supernatant, washing once, heat eluting, and adding the elution back to freshly immobilized protein for a total of three cycles. Elutions from the selections were prepped accordingly for sequencing using an Illumina MiniSeq. Data from the MiniSeq was then processed using Pipeline Pilot by BIOVIA and analyzed using Spotfire by TIBCO (see Supplemental for details).

In the sequencing data from both of the selections with CBX7, the positive control compound appeared at the top with dramatic enrichment with 8570 and 5308 reads respectively (Table 2, Figure S2). This is in relation to the frequency of 6 and 1 in sequencing data of the library without a selection. Of the 935 library member sequences identified in the sequencing data for experiment 1 with CBX7, the positive control accounted for over 80% of the total reads compared to 0.005% of the total reads in the library alone. For the duplicate experiment, the positive control only had a frequency of 1 in the library alone, falling into the majority of the distribution of compounds (Figure S3), but after the selection it reached 5308 reads. The negative control was not selected for in the CBX7 selection experiments and the negative control barcode was not found in the sequencing data. It should be noted that the depth of sequencing we obtained did not cover the entirety of our library members for the DEL alone. In addition to the selection described previously, we also completed a selection experiment involving only one incubation but with 5 washes (CBX7 short). The positive control remained at the top of the sequencing data, but with much less enrichment as it only produced a frequency of 117 (Table 2). When completing a selection with the Dynabeads alone, the positive control did appear at the top of the data; however, we believe this may be due to non-specific interactions as it is at a frequency with background levels at 0.6% of the total reads and evidence of the next best compounds having similar frequencies (Table S1). From these experiments we have verified that we successfully selected a positive control molecule spiked into a DEL and have set a benchmark for approximate anticipated reads when selecting a compound from a DEL with a K_d_ ~ 100 nM (UNC3866 K_d_ by ITC) [56].

## 3. Materials and Methods

### 3.1. Materials

Standard reagents and solvents were purchased from commercial sources. Fmoc protected building blocks were obtained from Sigma-Aldrich (St. Louis, MO, USA), Combi-Blocks (San Diego, CA), ChemBridge (San Diego, CA, USA), Alfa Aesar (Ward Hill, MA, USA), and Matrix Scientific (Columbia, SC, USA). Dynabeads^®^ MyOne™ Streptavidin C1 by Invitrogen were obtained from ThermoFisher Scientific (catalog number 65001). A Phusion High-Fidelity Polymerase PCR kit (catalog number E0553L), T4 DNA Ligase (catalog number M0202M), and DNA Polymerase I, Large (Klenow) Fragment (catalog M0210M) were obtained from New England Biolabs, Inc. (Ipswich, MA, USA). SsoAdvanced™ Universal SYBR^®^ Green Supermix (catalog number1725271) and Micro Bio-Spin™ P-30 Gel Columns (catalog number 7326202) were purchased from Bio-Rad Laboratories, Inc. (Hercules, CA, USA). The DNA scaffold (5′-/5Phos/GAGTCA/iSp9/iUniAmM/iSp9/TGACTCCCC-3′) and the DNA oligos used for tags were obtained from IDT, Inc., (Coralville, IA, USA). The SeQuant ZIC-pHILIC column (Part No: 2812-152) was purchased from MilliporeSigma (Burlington, MA, USA). NAP-5 columns by illustra (catalog number 17085302) were purchased from GE Healthcare LifeSciences (Pittsburgh, PA, USA) The Illumina sequencing consumables were purchased from Illumina (San Diego, CA, USA). Trizma^®^ hydrochloride, Sodium chloride, and Tween 20 were obtained from Sigma-Aldrich. 1,4-Dithio-DL-threitol (DTT) was obtained from Akron Biotech.

### 3.2. Methods

Detailed methods for building block validation, library construction, barcoded control compound production, sequencing, and data analysis can be found in the Supplementary Materials.

## 4. Conclusions and Future Directions

A focused DEL screening platform is an attractive option for hit discovery within a specific target class. It balances the ability to create a focused library, while still being able to explore a large chemical space. The DEL summarized in this communication has been produced as a target class focused library for Kme readers with multivalent characteristics. By taking a rational, structure-based approach to library design, our hypothesis is that a DEL screen using smaller libraries like the one reported here (such as <100,000 compounds) will produce hits suitable for chemical probe development of Kme readers and establish a preliminary SAR profile. This approach may reduce or bypass the need for substantial medicinal chemistry optimization and detailed structure-activity relationships, which can be especially challenging to develop for multivalent targets [73]. Overall, we anticipate that the library type we designed and report here will enable rapid hit generation for many Kme reader proteins with multiple modified-lysine binding pockets.

In future projects, automated computational workflows may play a more significant role in the screening follow-up process. They will be particularly helpful in prioritizing true hits for resynthesis and confirmation, exploring hit SAR, and hit-to-lead progression. For instance, machine learning techniques, including deep learning, might be used to increase the signal-to-noise ratio in the screening data and thus reduce the false positive rate among resynthesis candidates. At later stages, structure-based strategies can be applied to further improve the ligands’ affinity to the target. We believe the application of DEL technology to chromatin reader proteins that are multivalent can be explored to unlock new opportunities for basic, translational, and drug discovery research.

## Figures and Tables

**Table 1:** Examples of proteins with multivalent post-translational modification (PTM) recognition including a methyl-lysine (Kme) domain.

Protein	UniProt Accession	Domains	PTM Recognition	PDB ID	Disease Relevance	References
SETDB1	Q15047	Triple Tudor	H3K9me2/3K14ac	6BHD, 6BHE, 6BHI	Huntington’s, Cancer – Breast, Liver, Prostate	[3,25,26,27,28,29]
UHRF1	Q96T88	PHD, Tandem Tudor	H3(N-terminus)K9me3	4GY5	Cancer – Breast, Lung, Prostate, Liver	[4,30,31,32,33,34,35]
TRIM24	O15164	PHD, Bromo	H3K9K23ac	3O34	Cancer – Breast, Liver, Lung, Colon	[5,36,37,38,39]
TRIM33	Q9UPN9	PHD, Bromo	H3K9me3K14ac, H3K9me3K14acK18ac	3U5N, 3U5O	Cancer – Liver, Pancreatic, Leukemia	[6,37,40,41]
PHF8	Q9UPP1	PHD, Jumonji	H3K4me3K9me2	3KV4	Cancer – Prostate, Lung, Breast, Gastric	[7,42,43,44,45,46]
RAG2	P55895	PHD	H3R2meK4me3	2V85	Lymphoid tumors and immunological disorders	[8,47,48]
53BP1	Q12888	Tandem Tudor	*p53K381acK382me2	4X34	Cancer – Breast, Ovarian, Colon	[9,49,50,51,52]

*Non-histone PTMs.

**Table 2:** Sequencing results from experiments containing control compounds.

Input Library/ Experiment Sequenced	Number of Compound Barcodes	Total Reads	Positive Control Frequency	Negative Control Frequency
UNCDEL003 with controls, Prep 1	45,202	120,038	6	0
CBX7 with DEL Prep 1	935	10,570	8570	0
CBX7(short) with DEL Prep 1	47,933	152,754	117	0
MyOne Dynabeads with DEL Prep 1	1,145	5847	35	0
UNCDEL003 with controls, Prep 2	30,989	53,239	1	0
CBX7 with DEL Prep 2	1236	7460	5308	0

## Supplementary Materials

The following are available online at https://www.mdpi.com/1420-3049/25/4/979/s1, Methods for: Protein Expression and Purification, Analytical Methods, Building Block Validation, Ethanol Precipitation, DNA-Encoded Library Tags, UNCDEL003 Construction, Selections, Amplification and Sequencing, Data Analysis, Barcoded Control Compound Synthesis, qPCR experiments, and Building Blocks utilized in UNCDEL003.
