# Supplementary material for: Design and Construction of a Focused DNA-Encoded Library for Multivalent Chromatin Reader Proteins
Source: Molecules. 2020 Feb 22;25(4):979. doi: 10.3390/molecules25040979 (PMC7070942; doi:10.3390/molecules25040979)
Supplement: Supplementary file 1 [file molecules-25-00979-s001.pdf]

Molecules
DNA-Encoded Libraries Special Edition

Supplemental for:

Communication

Design and Construction of a Focused DNA-Encoded Library for Multivalent Chromatin Reader Proteins

Justin M. Rectenwald [1], Shiva Krishna Reddy Guduru [2], Zhao Dang [2], Leonard B. Collins [3], Yi-En Liao [2], Jacqueline L. Norris-Drouin [2], Stephanie H. Cholensky [2], Kyle W. Kaufmann [4], Scott M. Hammond [4], Dmitri B. Kireev [2], Stephen V. Frye [2*], and Kenneth H. Pearce [2*]

[1] UNC School of Medicine, Department of Biochemistry and Biophysics; jmr5808@email.unc.edu
[2] UNC Eshelman School of Pharmacy, Division of Chemical Biology and Medicinal Chemistry, Center for Integrative Chemical Biology and Drug Discovery; shivag@email.unc.edu, dangzhaoo@gmail.com, yienliao@live.unc.edu, jnorris@unc.edu, cholensk@email.unc.edu, dmitri.kireev@unc.edu, svfrye@email.unc.edu, khpearce@unc.edu
[3] UNC Gillings School of Public Health, Biomarker Mass Spectrometry Facility; lbcollin@email.unc.edu
[4] UNC School of Medicine, Department of Cell Biology and Physiology; kyle_kaufmann@med.unc.edu, scott_hammond@med.unc.edu
University of North Carolina at Chapel Hill, Chapel Hill, NC. 27599

* Correspondence: svfrye@email.unc.edu (S.V.F); khpearce@unc.edu (K.H.P)

Table of Contents

- *Main Text Referenced Supplemental Figures and Table*
- *Protein Expression and Purification*
- *Analytical Methods*
- *Building Block Validation*
- *Ethanol Precipitation*
- *DNA-Encoded Library Tags*
- *UNCDEL003 Construction*
- *Building Blocks Utilized in UNCDEL003*
- *Selections*
- *Amplification and Sequencing*
- *Data Analysis*
- *Barcoded Control Compound Synthesis*
- *qPCR experiments*

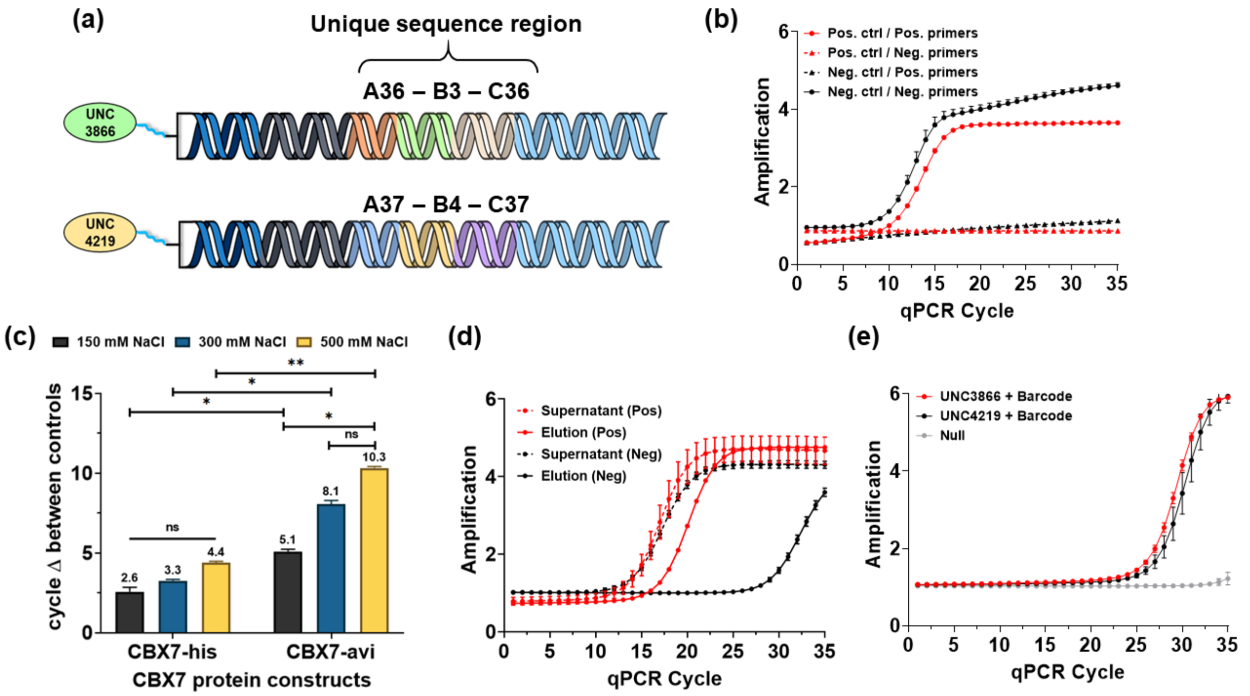

Figure S1. Examples of qPCR experiments for selection optimization using barcoded control compounds. (a) Illustration of controls with unique barcode regions and the tags used. (b) Selective amplification of barcoded controls as demonstrated by qPCR. (c) Comparison of investigative selection conditions using barcoded control compounds. Shown is the difference in cycle time each compound amplifies for respective conditions. Significance was determined using the student's t-test (ns, $P > 0.05$; *, $P \leq 0.05$; **, $P \leq 0.01$; ***, $P \leq 0.001$). (d) Example qPCR amplification traces of the CBX7-avi selection completed using 500 mM NaCl in the buffer. (e) qPCR of UNCDEL003 control compounds to show comparable amplification. All qPCR experiments were completed in duplicate.

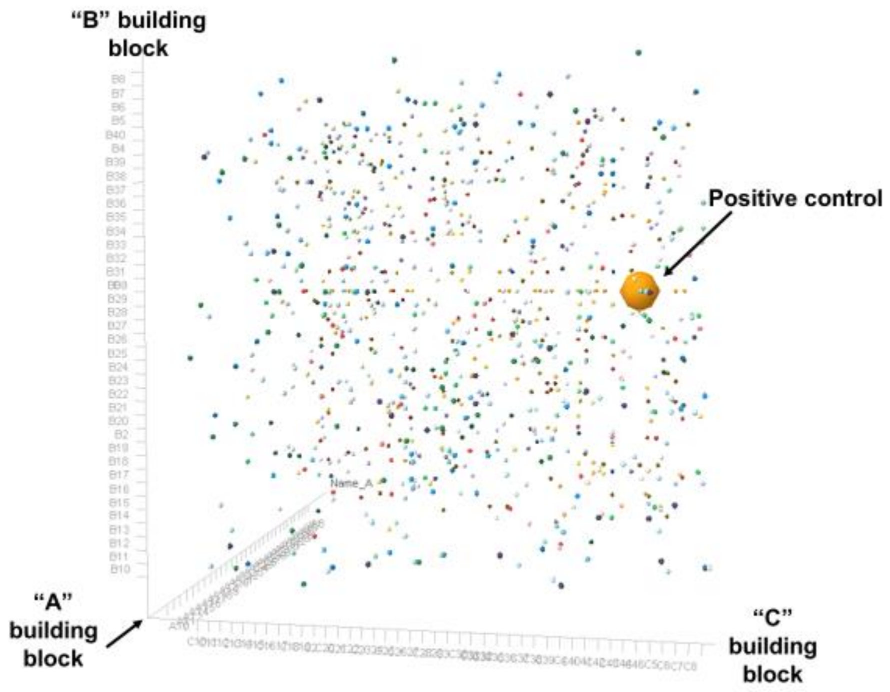

Figure S2. A three-dimensional plot showing the dramatic enrichment of the positive control compound versus the rest of the compounds that were sequenced in the first CBX7 selection experiment.

Table S1: Values for top 30 sequences found in the MyOne Dynabeads only selection

ID	Frequency	Rank	cRank
A36-B3-C46 (Positive Control)	35	1	1
A26-B16-C22	29	2	2
A26-B14-C3	27	3	3
A10-B14-C29	26	4	4
A31-B24-C34	26	5	4
A31-B31-C13	26	6	4
A10-B39-C27	25	7	5
A17-B39-C40	25	8	5
A15-B39-C15	25	9	5
A8-B40-C18	24	10	6
A10-B15-C11	24	11	6
A14-B31-C20	24	12	6
A27-B15-C33	24	13	6
A8-B15-C26	24	14	6
A9-B34-C26	23	15	7
A31-B10-C24	23	16	7
A10-B17-C4	22	17	8
A23-B16-C34	22	18	8
A14-B27-C32	22	19	8
A7-B7-C9	22	20	8
A26-B20-C19	22	21	8
A10-B16-C18	22	22	8
A28-B21-C2	22	23	8
A18-B27-C3	21	24	9
A12-B11-C14	21	25	9
A31-B24-C24	21	26	9
A10-B31-C29	21	27	9
A8-B19-C22	21	28	9
A8-B16-C41	21	29	9
A29-B11-C33	20	30	10

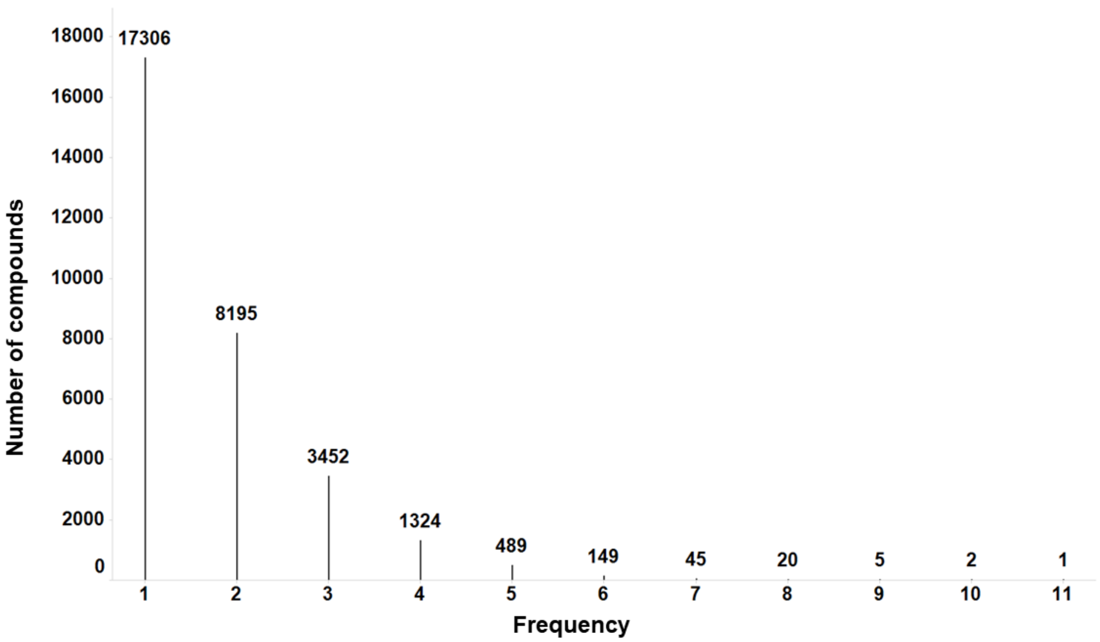

Figure S3. Distribution of frequencies for UNCDEL003 (experiment 2) with spike-in controls. Exact number of compounds at each frequency are shown above lines, The CBX7 positive control had a frequency of 1.

Protein Expression and Purification

The chromodomain of CBX7 (residues 8–62 of NP_783640) was expressed with either a C-terminal His-tag in a pET30 expression vector or a C-terminal Avi-tag in a modified pET28 expression vector. This modified pET28 vector includes both an N-terminal His-tag and C-terminal Avi-tag sequence (GLNDIFEAQKIEWHE).

All expression constructs were transformed into Rosetta 2 BL21(DE3)pLysS competent cells (Novagen, EMD Chemicals, San Diego, CA). Protein expression was induced by growing cells at 37 °C with shaking until the OD_{600} reached ~0.6–0.8 at which time the temperature was lowered to 18 °C and expression was induced by adding 0.5 mM IPTG with shaking continuing overnight. Biotinylated CBX7 was produced by co-transforming BL21 cells with an expression plasmid for E. coli biotin ligase, BirA (pET21a-BirA was a gift from Alice Ting (Addgene plasmid # 20857), Howarth et al., 2005) and adding D-biotin to a final concentration of 50 µM at the same time as IPTG. Cells were harvested by centrifugation and pellets were stored at -80°C.

Proteins were purified by re-suspending thawed cell pellets in 30 mL of lysis buffer (50 mM sodium phosphate pH 7.2, 50 mM NaCl, 30 mM imidazole, 1× EDTA free protease inhibitor cocktail (Roche Diagnostics, Indianapolis, IN)) per liter of culture. Cells were lysed on ice by sonication with a Branson Digital 450 Sonifier (Branson Ultrasonics, Danbury, CT) at 40% amplitude for 12 cycles with each cycle consisting of a 20 s pulse followed by a 40 s rest. The cell lysate was clarified by centrifugation and loaded onto a HisTrap FF column (GE Healthcare, Piscataway, NJ) that had been pre-equilibrated with 10 column volumes of binding buffer (50 mM sodium phosphate, pH 7.2, 500 mM NaCl, 30mM imidazole) using an AKTA FPLC (GE Healthcare, Piscataway, NJ). The column was washed with 15 column volumes of binding buffer and protein was eluted in a linear gradient to 100% elution buffer (50 mM sodium phosphate, pH 7.2, 500 mM NaCl, 500 mM imidazole) over 20 column volumes. Peak fractions containing the desired protein were pooled and concentrated to 2 mL in Amicon Ultra-15 concentrators 3,000 molecular weight

cut-off (Merck Millipore, Carrigtwohill Co. Cork IRL). Concentrated protein was loaded onto a HiLoad 26/60 Superdex 75 prep grade column (GE Healthcare, Piscataway, NJ) that had been pre-equilibrated with 1.2 column volumes of sizing buffer (25 mM Tris, pH 7.5, 250 mM NaCl, 2 mM DTT, 5% glycerol) using an ATKA Purifier (GE Healthcare, Piscataway, NJ). Protein was eluted isocratically in sizing buffer over 1.3 column volumes at a flow rate of 2 mL/min collecting 3-mL fractions. Peak fractions were analyzed for purity by SDS-PAGE and those containing pure protein were pooled and concentrated using Amicon Ultra-15 concentrators 3,000 molecular weight cut-off. Concentrated C-His tagged protein was aliquoted and stored at -80˚C in sizing buffer while C-Avi tagged protein was further purified as described below.

Biotinylated CBX7 was isolated by passing concentrated C-Avi tagged protein over a 2 mL monomeric avidin column by gravity flow following manufacturer's recommendations (Pierce Biotechnology/Thermo Scientific, Rockford, IL). Bound protein was eluted in 12 mL PBS containing 2 mM D-biotin. The N-terminal His-tag was removed from biotinylated CBX7 by thrombin cleavage according to manufacturer's recommendations (Novagen, EMD Chemicals, San Diego, CA). Briefly, purified protein was incubated with thrombin at a final concentration of 1 unit thrombin per milligram tagged protein for 16 hours at 4 ^{0}C. The cleavage reaction was then passed over a HisTrap FF column to remove any protein that still retained the tag, followed by size exclusion over a Superdex 75 column to remove thrombin as described above. Peak fractions were analyzed for purity by SDS-PAGE and those containing pure protein were pooled and concentrated using Amicon Ultra-15 concentrators 3,000 molecular weight cut-off. Concentrated protein was aliquoted and stored at -80°C in sizing buffer.

Reference for the BirA expression plasmid:

Howarth, M.; Takao, K.; Hayashi, Y.; et al. Targeting Quantum Dots to Surface Proteins in Living Cells with Biotin Ligase. *Proc. Natl. Acad. Sci. U. S. A.* **2005**, *102*, 7583–7588.

Analytical Methods

An Agilent Technologies 6520 Accurate-Mass Q-TOF LC-MS was used to analyze on-DNA reactions during building block validation and during library synthesis. Samples were injected onto a ZIC-*p*HILIC hydrophilic interaction chromatography column (5 µm, 2.1 x 150 mm) and eluted (25 – 90% solvent A) over 6 minutes with a hold for 4 minutes, 0.2 mL/min flow rate with monitoring at 260 nm; Solvent A: 100 mM ammonium bicarbonate in deionized water; Solvent B: 50% acetonitrile/50% deionized water. Effluent was analyzed on an electrospray mass spectrometer in positive ion mode, in standard mass range (3200 *m/z*), and with extended dynamic range (2 *GHz*).

Table S2: LC-MS solvent gradient

Time (min)	A (%)	B (%)	Flow [mL/min]	Max Pressure Limit [bar]
0.00	25	75	0.200	600
6.00	90	10	0.200	600
10.00	90	10	0.200	600
12.50	25	75	0.200	600
16.00	25	75	0.200	600

Building Block Validation

Building blocks were tested using the similar conditions to those used for the library synthesis. Reaction yields were characterized through examination of the TIC traces in the LC-MS chromatograms. Detection at absorbance of 260 nm (A260) was also utilized to track the DNA conjugates. In general, reaction products are detected between 7.5 and 7.9 minutes. Building blocks which gave >80% yield in the reactions with little starting material were used for the library. Reactions of building blocks that produced side products were excluded from the library as they would make deconvolution more difficult. In total, 128 building blocks were tested throughout the development process. 27 building blocks did not meet the described requirements for library incorporation. Not every building block was incorporated into the library due to compound availability.

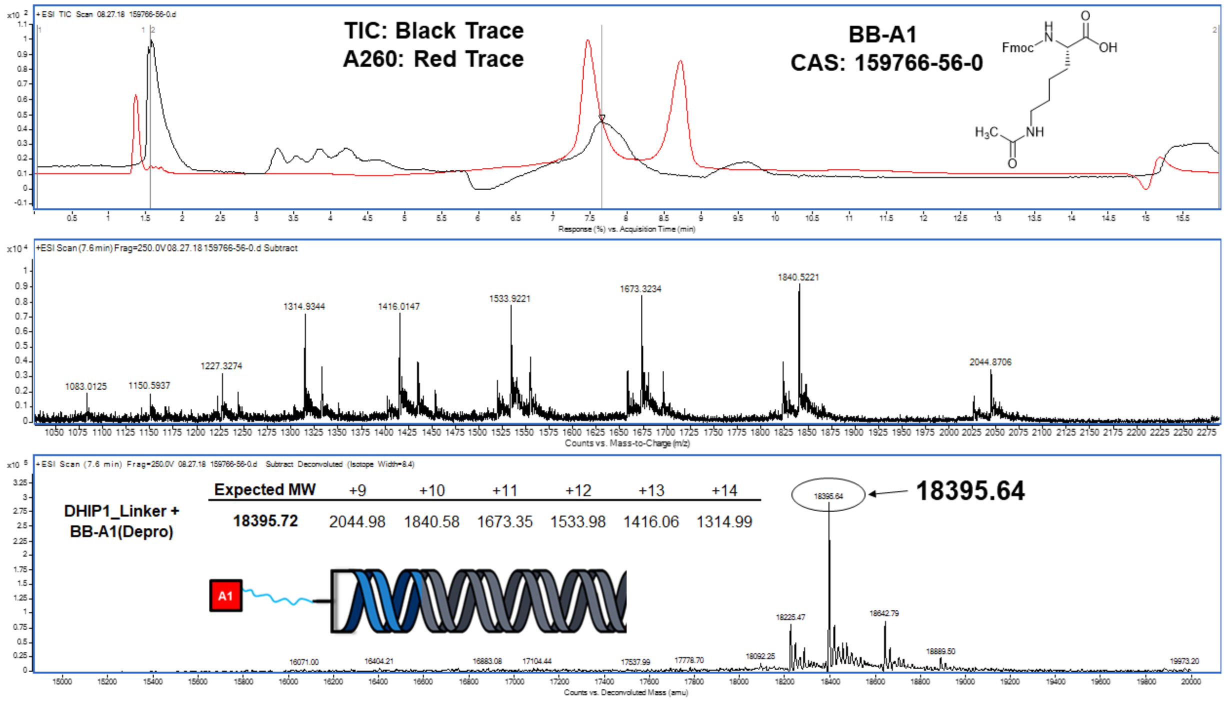

Figure S4. Shown is an example of a building block that coupled successfully and meets the described requirements for library incorporation. Note: The peak of the A260 trace at 8.7 minutes is due to non-specific DNA remaining on the column.

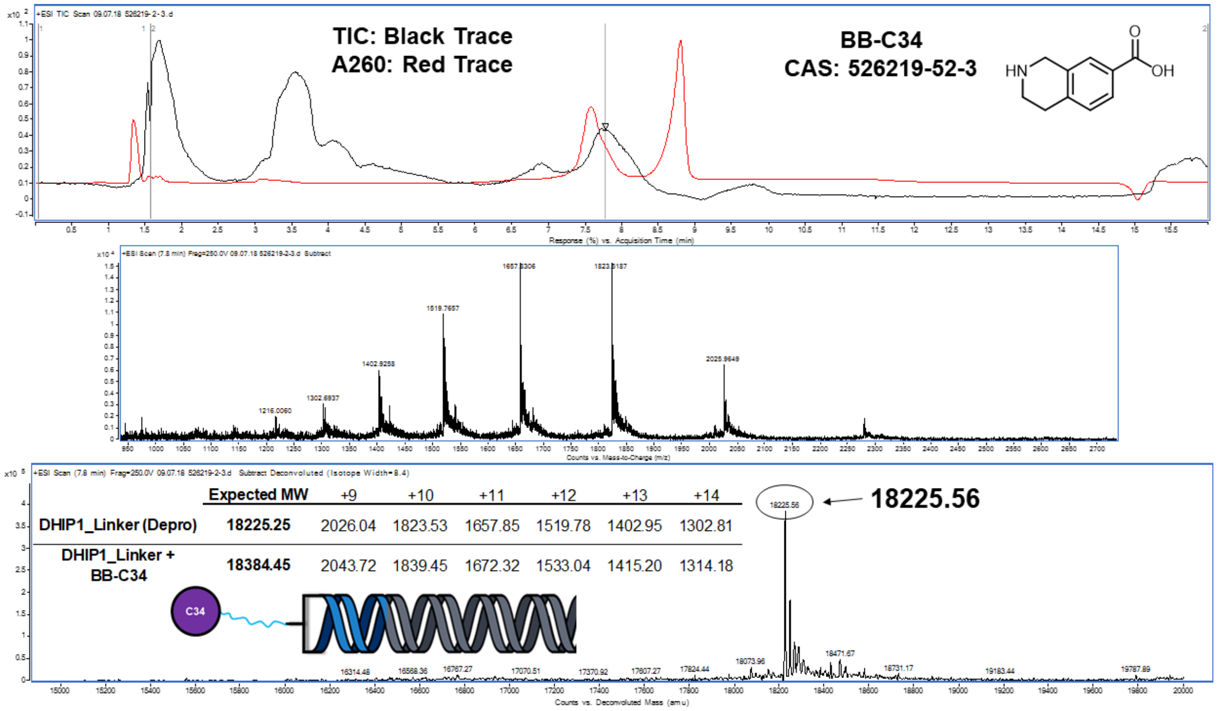

Figure S5. Shown is an example of a building block that did not meet requirements. The major DNA conjugate is the starting material. Note: The peak of the A260 trace at 8.8 minutes is due to non-specific DNA remaining on the column.

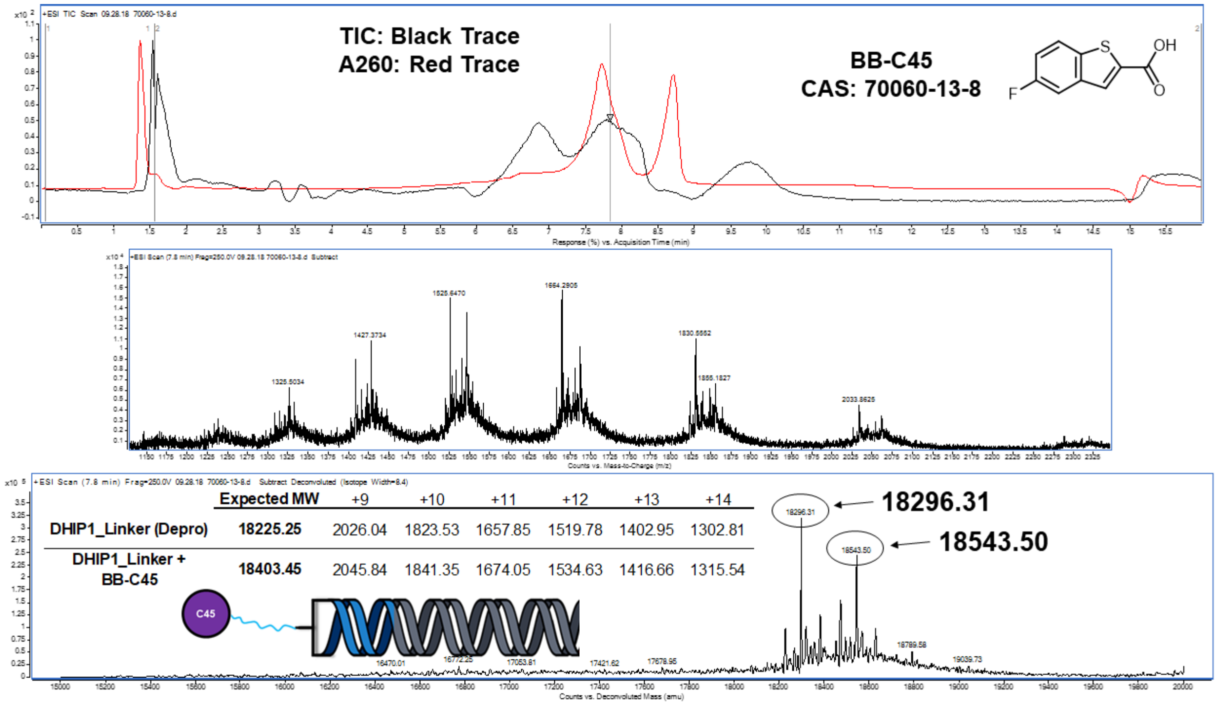

Figure S6. Shown is an example of building block that did not meet requirements because a different product than expected was formed. Note: The peak of the A260 trace at 8.6 minutes is due to non-specific DNA remaining on the column.

Building blocks that meet requirements for library incorporation

CAS: 252049-10-8 | CAS: 201004-29-7 | JHL002-030 | VAH001-005b | JW001-001 | CAS: 159766-56-0 | CAS: 201004-23-1 | CAS: 132307-50-7 | IE003-012-1

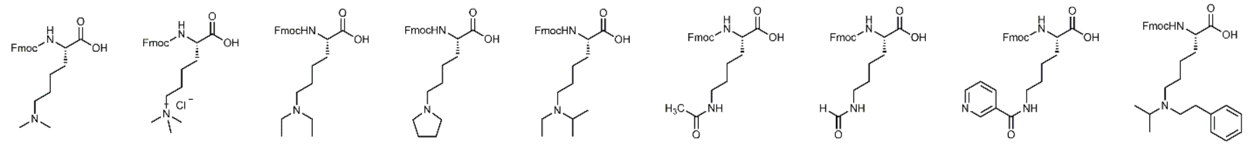

KDB001 | CAS: 959578-11-1 | CAS: 112883-43-9 | CAS: 199110-64-0 | CAS: 84030-19-3 | CAS: 35737-15-6 | GSKR-001-02b | GSKR-001-11

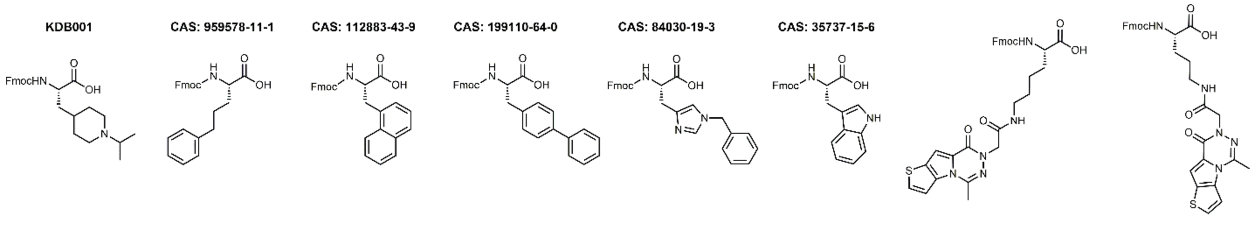

CAS: 269078-71-9 | CAS: 175453-07-3 | CAS: 159611-02-6 | CAS: 135944-07-9 | CAS: 86123-10-6 | CAS: 214750-76-2 | CAS: 204322-23-6 | CAS: 169243-86-1

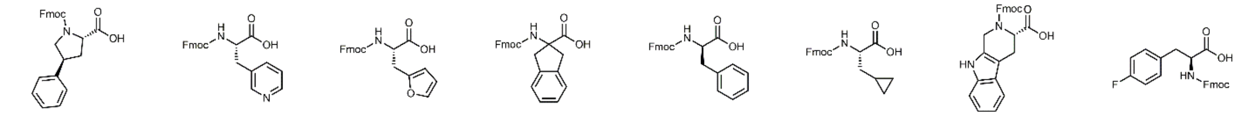

CAS: 35737-10-1 | CAS: 29022-11-5 | CAS: 116821-47-7 | CAS: 260367-12-2 | CAS: 166108-71-0 | CAS: 872679-70-4 | CAS: 669073-62-5

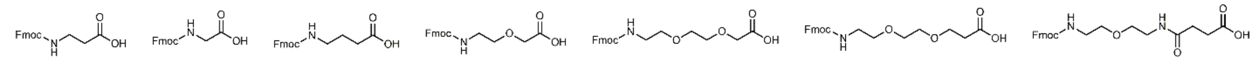

CAS: 88574-07-6 | CAS: 557756-85-1 | ZD0107 G1R | CAS: 148928-15-8 | CAS: 147900-46-7 | CAS: 193693-68-4

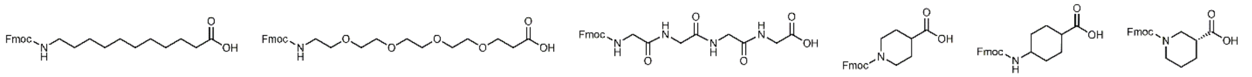

CAS: 180576-05-0 | CAS: 136030-33-6 | CAS: 150256-42-1 | CAS: 185116-42-1 | CAS: 193693-64-0 | CAS: 136552-06-2 | CAS: 28524-99-6 | CAS: 122996-47-8 | CAS: 130309-37-4

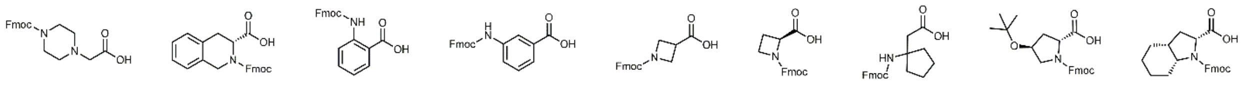

CAS: 117322-30-2 | CAS: 185116-43-2 | CAS: 164470-64-8 | CAS: 186320-14-9 | CAS: 215248-47-8 | CAS: 269078-84-4 | CAS: 882847-24-7 | CAS: 946716-21-8

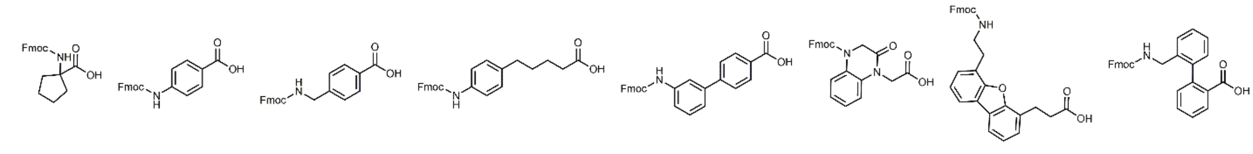

CAS: 22090-27-3 | CAS: 34798-68-0 | CAS: 510723-50-9 | CAS: 316833-32-6 | CAS: 106261-49-8 | CAS: 914637-18-6 | CAS: 95124-68-8 | CAS: 499-86-5

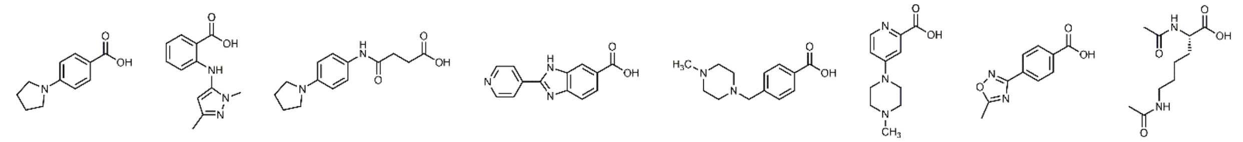

CAS: 71953-90-7 | ZD-01-27 | CAS: 94108-46-0 | CAS: 141943-04-6 | CAS: 3025-96-5 | CAS: 37386-15-5 | CAS: 556-08-1 | CAS: 619-84-1 | CAS: 456-22-4

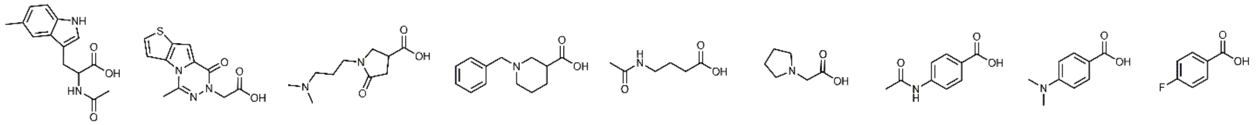

CAS: 459-31-4 | CAS: 80875-98-5 | CAS: 4865-84-3 | CAS: 87-51-4 | CAS: 53440-12-3 | CAS: 214848-62-1 | CAS: 956317-36-5 | CAS: 3093-97-8 | CAS: 98-98-6

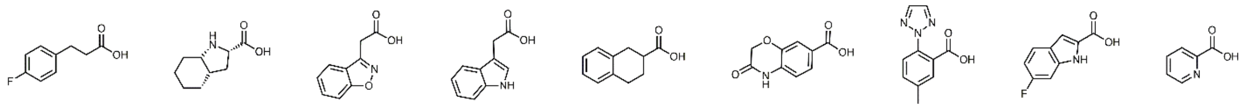

CAS: 23814-14-4 | CAS: 15788-16-6 | CAS: 2861-28-1 | CAS: 1560-11-8 | CAS: 931-03-3 | CAS: 14527-41-4 | CAS: 1072-84-0 | CAS: 1072-84-0 | CAS: 25023-36-3

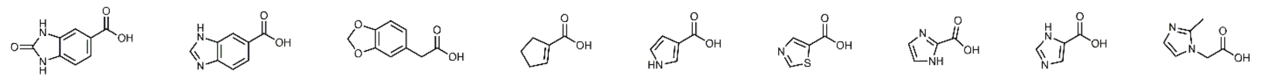

CAS: 527-72-0 | CAS: 455-38-9 | CAS: 455-40-3 | CAS: 543-24-8 | CAS: 4854-46-0 | CAS: 68947-43-3 | CAS: 54699-92-2 | CAS: 85064-61-5 | CAS: 122665-97-8

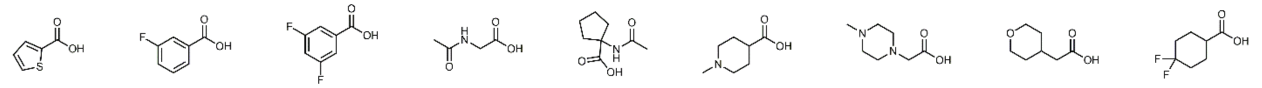

CAS: 35661-40-6 | CAS: 312965-05-2

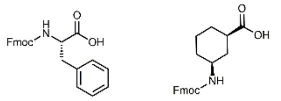

Building blocks that do not meet requirements for library incorporation

Sigma-Aldrich: CDS020833 | CAS: 374899-60-2 | VAH001-012 | KDB00276 | KDB001-2 | JHL003-35 | CAS: 77284-32-3 | CAS: 221352-82-5 | Sigma-Aldrich: MFCD00273479

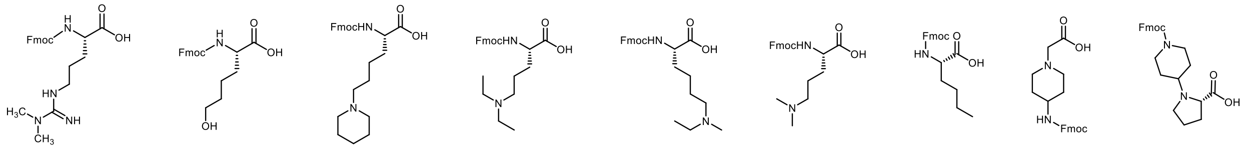

CAS:198560-68-8 | CAS: 175453-07-3 | CAS: 205528-32-1 | Sigma-Aldrich: MFCD01632028 | ZD-01-10 | CAS: 176504-01-1

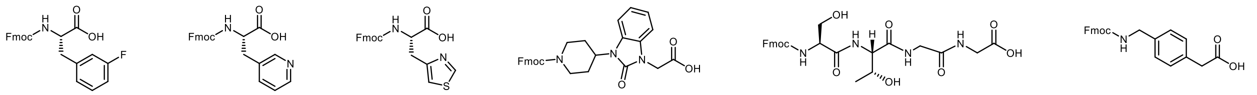

Sigma-Aldrich: MFCD01074698 | CAS: 526219-52-3 | CAS: 498-94-2 | CAS: 15966-72-0 | CAS: 99361-29-2 | CAS: 79888-39-4 | CAS: 4052-30-6 | CAS: 70060-13-8

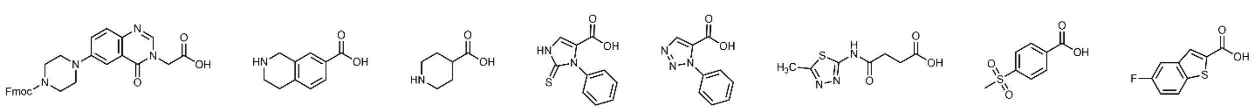

CAS: 66937-99-3 | CAS: 3235-67-4 | CAS: 98-78-2 | CAS: 16179-97-8

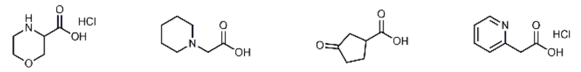

Ethanol Precipitation

To a DNA product, 10% by volume of 5 M NaCl was added, followed by 2.5 volumes of cold ethanol before placing in a −80 °C overnight. The sample was then centrifuged at 14,000xg for 4 hours at 4 °C. The resulting supernatant was discarded and the pellet was rinsed once with cold 70% ethanol (200 μL). After centrifugation at 14,000xg for another 30 minutes at 4 °C, the supernatant was discarded and the pellet was dried on a speed-vac for 60 minutes.

DNA-Encoded Library Tags

<u>DEL-scaffold (5′-/5Phos/GAGTCA/iSp9/iUniAmM/iSp9/TGACTCCCC-3′)</u>

5' TGACTCCCC 3'
3' ACTGAG 5'

<u>Initial Primer Sequence</u>

5' TGGCGACCGATGCCGCCAAG 3'
3'GGGACCGCTGGCTACGGCGGT 5'

Cycle tags contain an eight-base pair coding region with two-base 3′ overhangs on each stand. For the ligations, the 5′ ends are phosphorylated. The two-base overhangs are invariant so the ligations from each cycle can proceed more efficiently and to ensure tags ligate to the previous cycle specifically.

	Cycle 1, "A" Tags	*Cycle 2, "B" Tags*	*Cycle 3, "C" Tags*	
5'	XXXXXXXXGT	XXXXXXXXGA	XXXXXXXXTT	3'
3'	TCXXXXXXXX	CAXXXXXXXX	CTXXXXXXXX	5'

<u>Closing Primer Sequence</u>

5' ACGGTCATGC 3'
3' AATGCCAGTACG*NNNNNNN*CGATCAGGCAGTCATCGG 5'

One strand of the Closing Primer contains a random, seven-base pair region which acts as a unique molecular identifier (UMI) for individual molecules in the library. The variable region is useful for eliminating PCR duplication during sequencing data analysis.

<u>UNCDEL003 Production</u>

Ligation of Initial Primer

To the DEL-scaffold (95 μM, 332 μL) was added 50 μL of 885 μM Initial Primer solution (1.4 equivalents), followed by 58 μL molecular biology (MB) grade water, 50 μL of 10X ligation buffer, and 10 μL T4 DNA ligase (2000 U/μL). This was completed in quadruple. The ligation was incubated at room temperature for 4 hours on a tabletop shaker. The ligations were quenched at 65 °C for 10 minutes. The ligation mixtures were combined and dried on a speed-vac. The pellet was resuspended into 500 μL of MB grade water and cleaned up using a GE NAP-5 size exclusion column. The DNA product was dried once more using a speed-vac in order to concentrate the sample. The DNA construct was resuspended into 100 μL of MB grade water.

Coupling of PEG-Linker

To several solutions of 2000 pmol DNA-scaffold-initial primer in coupling reaction buffer (50 mM MOPS, pH 8.0, 0.5 M NaCl, 96 μL) was added a mixture of Fmoc-15-amino-4,7,10,13-tetraoxapentadecanoic acid (60 mM, 60 μL), EDC (300 mM, 12 μL), HOAt (60 mM, 12 μL) and DIPEA (300 mM, 24 μL) in DMSO, previously activated for 15 minutes at room temperature. The reactions were agitated at room temperature for 18 hours. A second addition of freshly activated acid in DMSO was prepared the same as before, then added to the reaction. The coupling was agitated for 6 additional hours at room temperature. The reactions were quenched using NH_4OAc (500 mM, 36 μL) and agitated for 30 minutes at room temperature. The DNA was isolated by ethanol precipitation. The DNA-pellets were then subjected to 50 μL of 20% piperidine/water for two hours. The deprotected samples were combined into a single tube, dried on a speed-vac, and resuspended into 500 μL of MB grade water. NAP-5 columns were used to remove excess reagents and salts from the sample. The desalted sample was dried again using a speed-vac to concentrate. The sample was resuspended into 100 μL of molecular biology grade water. The final combined yield was 94,000 pmol of material.

Cycle 1

2750 pmol of DNA-headpiece + Initial Primer + PEG-linker was added to 33 tubes. To each tube was added a corresponding "A" tag at 1.4 equivalents. Total reaction volumes were 50 μL with 2 μL of T4 DNA ligase. Ligations were left for 4 hours at room temperature. The ligations were quenched at 65 °C for 10 minutes on a heat block, then allowed to cool to room temperature on the benchtop. The ligation products were purified using BioRad P30 micro-spin columns. Yields for all ligations after the spin column were > 75%. Volumes of each sample were brought to 96 μL using coupling reaction buffer (50 mM MOPS, pH 8.0, 0.5 M NaCl). "A" building blocks (60 μL of 60 mM FMOC-protected acids,~1800 eq.) were activated using 12 μL of 300 mM EDC-HCl, 12 μL of 60 mM HOAt, and 24 μL of 300 mM DIPEA for 15 minutes at room temperature. The activated building blocks were then added to the appropriate "A" tagged DNA-constructs. Reactions proceeded for 18 hours at room temperature on a tabletop shaker. A second addition of activated acid, using the same volumes, was added to the reactions and allowed to proceed for 6 more hours. The reactions were quenched by addition of NH_4OAc (500 mM, 36 μL) at room temperature for 30 minutes and the DNA was isolated by ethanol precipitation. The pellets from the ethanol precipitation were subjected to 200 μL of 10% piperdine/water for two hours, then dried on a speed-vac. The pellets were resuspended into 50 μL of molecular biology water and desalted using BioRad P-30 micro-spin columns. On average, yields were > 80% for each reaction. 1500 pmol of each reaction product was combined and taken for cycle 2.

Cycle 2

1167 pmol of cycle 1 pool was placed into 40 Eppendorf tubes. To each tube was added 1.4 equivalents of a corresponding "B" tag. The total reaction volume of each ligation was 50 μL with 2 μL of T4 DNA ligase being used. The ligations proceeded for 4 hours at room temperature before being quenched for 10 minutes at 65 °C on a heat block. Once cooled, the ligation products were purified using a BioRad P30 micro-spin column. Yields for each ligation measured after the spin columns were > 90%. The volume of each sample was brought to 96 μL using coupling reaction buffer (50 mM MOPS, pH 8.0, 0.5 M NaCl). "B" building blocks (60 μL of 60 mM FMOC-protected acids,~3000 eq.) were activated using 12 μL of 300 mM EDC-HCl, 12 μL of 60 mM HOAt, and 24 μL of 300 mM DIPEA for 15 minutes at room temperature,

then added to the appropriate DNA-tagged constructs. The coupling reactions were left at room temperature for 18 hours on a tabletop shaker. A second addition of activated acid was added to the mixture and left for another 6 hours. Addition of NH_4OAc (500 mM, 36 µL) was used to quench the reactions by at room temperature for 30 minutes and the DNA was isolated by ethanol precipitation. 200 µL of 10% piperdine/water was added to the pellets from the ethanol precipitation for two hours, then dried on a speed-vac. The deprotected products were resuspended into 50 µL of molecular biology water and desalted using BioRad P-30 micro-spin columns. On average, yields were > 50% for each reaction. 700 pmol of each reaction product was combined and taken for cycle 3.

Cycle 3

655 pmol of cycle 2 pool was placed into 44 Eppendorf tubes. To each tube was added 1.4 equivalents of a corresponding "C" tag. The total reaction volume of each ligation was 125 µL with 2 µL of T4 DNA ligase being used. The ligations proceeded for 24 hours at room temperature before being quenched for 10 minutes at 65 °C on a heat block. Once cooled, the ligation products were dried using a speed-vac. The volume of each sample was brought to 96 µL using coupling reaction buffer (50 mM MOPS, pH 8.0, 0.5 M NaCl). "C" building blocks (60 µL of 60 mM carboxylic acids,~5500 eq.) were activated using 12 µL of 300 mM EDC-HCl, 12 µL of 60 mM HOAt, and 24 µL of 300 mM DIPEA for 15 minutes at room temperature before adding to the DNA. The coupling reactions were left at room temperature for 18 hours on a tabletop shaker. A second addition of activated acid was added to the mixture and left for another 6 hours. Addition of NH_4OAc (500 mM, 36 µL) was used to quench the reactions by at room temperature for 30 minutes and the DNA was isolated by ethanol precipitation. The pellets from the ethanol precipitation were dried on a speed-vac, then resuspended into 50 µL of molecular biology water and desalted using BioRad P-30 micro-spin columns. On average, yields were > 60% for each reaction. Approximately 350 pmol of each reaction product was combined as the cycle 3 product, with the final yield being 13,400 pmol of DNA.

Closing Primer Addition

Until ready for selections, the DEL was kept as a cycle 3 product. Before selections, two equivalents of Closing Primer were ligated to a 1000 pmol aliquot of library using T4 DNA ligase; ligation volume 50 µL. The ligation product was desalted using a BioRad P30 micro-spin column. The library Closing Primer was then filled by Klenow polymerization using DNA Polymerase I, Large (Klenow) Fragment overnight on the benchtop. The library was heat quenched at 80 °C for 20 minutes then allowed to cool to room temperature. The final library product was then desalted using a BioRad P30 micro-spin column.

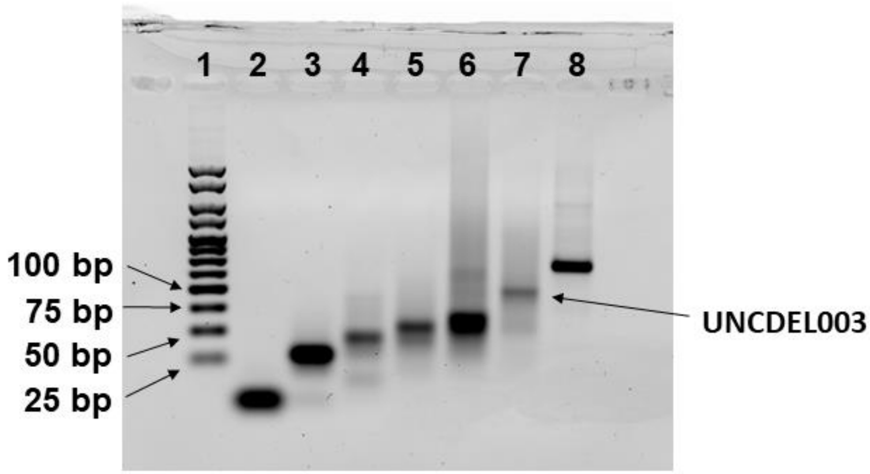

Figure S7. Agarose gel visualization of library steps; (1) 25 bp ladder (2) DNA Scaffold (3) DHIP003 (4) Combined Cycle 1 (5) Combined Cycle 2 (6) Combined Cycle 3 (7) UNCDEL003 (8) 35 Cycle PCR of UNCDEL003.

Table S3: Building blocks utilized for UNCDEL003

Cycle 1		Cycle 2		Cycle 3	
**Building Block**	**Cas # / Identifier**	**Building Block**	**Cas # / Identifier**	**Building Block**	**Cas # / Identifier**
A1	159766-56-0	B1	35737-10-1	C1	22090-27-3
A2	201004-23-1	B2	29022-11-5	C2	106261-49-8
A3	132307-50-7	B3	116821-47-7	C3	510723-50-9
A4	252049-10-8	B4	260367-12-2	C4	316833-32-6
A5	201004-29-7	B5	166108-71-0	C5	34798-68-0
A6	JW001-001	B6	872679-70-4	C6	914637-18-6
A7	JHL002-030	B7	669073-62-5	C7	95124-68-8
A8	VAH001-005b	B8	88574-07-6	C8	499-86-5
A9	IE003-012-1	B9	557756-85-1	C9	71953-90-7
A10	KDB001	B10	ZD0107 G1R	C10	ZD-01-27
A11	GSKR-001-02b	B11	148928-15-8	C11	94108-46-0
A12	GSKR-001-11	B12	147900-46-7	C12	141943-04-6
A13	204322-23-6	B13	193693-68-4	C13	3025-96-5
A14	199110-64-0	B14	132307-50-7	C14	37386-15-5
A15	269078-71-9	B15	180576-05-0	C15	556-08-1
A16	84030-19-3	B16	136030-33-6	C16	619-84-1
A17	959578-11-1	B17	185116-43-2	C17	456-22-4
A18	112883-43-9	B18	150256-42-1	C18	459-31-4
A19	35737-15-6	B19	185116-42-1	C19	80875-98-5
A20	86123-10-6	B20	164470-64-8	C20	4865-84-3
A21	175453-07-3	B21	186320-14-9	C21	87-51-4
A22	169243-86-1	B22	215248-47-8	C22	53440-12-3
A23	214750-76-2	B23	269078-84-4	C23	214848-62-1
A24	159611-02-6	B24	882847-24-7	C24	956317-36-5
A25	135944-07-9	B25	946716-21-8	C25	23814-14-4
A26	215248-47-8	B26	214750-76-2	C26	15788-16-6
A27	282524-99-6	B27	193693-64-0	C27	3093-97-8
A28	122996-47-8	B28	136552-06-2	C28	2861-28-1
A29	117322-30-2	B29	282524-99-6	C29	1560-11-8
A30	130309-37-4	B30	122996-47-8	C30	931-03-3
A31	269078-84-4	B31	117322-30-2	C31	14527-41-4
A32	193693-64-0	B32	130309-37-4	C32	16042-25-4
A33	136552-06-2	B33	135944-07-9	C33	1072-84-0
		B34	269078-71-9	C34	25023-36-3
		B35	204322-23-6	C35	527-72-0
		B36	169243-86-1	C36	98-98-6
		B37	159766-56-0	C37	455-38-9
		B38	VAH001-005b	C38	455-40-3
		B39	JHL002-030	C39	543-24-8
		B40	IE003-012-1	C40	4854-46-0
				C41	71985-80-3
				C42	54699-92-2
				C43	85064-61-5
				C44	122665-97-8

UNCDEL003 Selections

A buffer containing 20 mM Tris-HCl, pH 7.5, 300 mM NaCl, 0.05% Tween-20, and 2 mM DTT was used throughout. 1 μM of biotinylated protein was immobilized on 2 μL Dynabeads MyOne Streptavidin C1 beads for 15 minutes (total volume 50 μL). Following immobilization, excess protein was washed away manually on a magnetic rack *via* three washes of 200 μL of buffer. UNCDEL003 (10 pmol in 100 μL) was added to the immobilized protein and incubated for one hour on a gentle, rotating mixer. After one hour, the supernatant was removed. One wash with 200 μL of buffer was performed. Fresh buffer was added to the protein/DEL mixture for a heat elution at 80 °C for 10 minutes. The elution was removed and added to a fresh batch of immobilized protein. The incubation cycle was completed for a total of three times.

Amplification and Sequencing

Following the elution, the remaining DNA was qPCR amplified using a BioRad Real Time PCR instrument. 50 μL reactions contained 1 μL of elution, 500 nM of each primer, 200 mM dNTPs, Phusion high-fidelity polymerase, Phusion high-fidelity buffer, and 0.100 μL of a 1:13,000 dilution of SYBR Green. Reactions followed the qPCR protocol: 5 min at 95 °C, then 35 cycles of 30 s at 92 °C; 15s at 55 °C; 15 s at 72 °C, followed by 10 min at 72 °C. The qPCR samples were stopped and removed 2 cycles post-plateau. Amplified DNA was purified using a PAGE system for DNA. Bands from the PAGE gel were extracted and ethanol precipitated. PAGE-purified DNA was sequenced using a 300 cycle Mid Output Reagent Cartridge on an Illumina MiniSeq instrument.

Data Analysis

Data files from the Illumina MiniSeq instrument were first converted from .bcl files to fastq.gz files using the bcl2fastq2 utility on the UNC Longleaf supercomputing cluster. Pipeline Pilot software was utilized to analyze sequence data in the text format. First, only lines that contained the DNA-scaffold and ClosingPrimer sequences were accepted. Then sequences from each cycle associated with building block addition were deconvoluted. Duplicate random regions of each Closing Primer of each particular compound were concatenated to one frequency count. The compounds were then ranked on sequence frequency. A second parameter, a cluster ranking, was given to each compound that shared the same frequency as another to prevent over ranking of a compound. Analysis of the processed data was visualized in Excel and in the software Spotfire, by TIBCO.

Control Compound Synthesis

General Solid-Phase Peptide Synthesis Procedures

CBX7 positive and negative control compounds synthesis was achieved through solid phase peptide synthesis on 2-Chlorotrityl chloride (2-CTC) resin. Peptide syntheses were performed in a polypropylene syringe fitted with a polyethylene porous frit. Solvents and soluble reagents were removed by applying pressure on flat top syringe piston. 2-CTC (0.2 mmol) resin was swollen in DCM (4 mL) for 30 min on a shaker. After removing the solvent, the syringe was filled with pre-activated Fmoc protected PEG4 amino acid in DCM (2 mL) allowed to shake for 12 hours. [Pre-activation of Fmoc-PEG4-amino acid(AA): To a solution of Fmoc-PEG4-AA (1 mmol) in DCM (2 mL) was added DIEA (1.2 mmol) followed by HATU coupling reagent (1 mmol), and the solution was allowed to sit for 10 min.] Then, the solvent was removed and the resin was washed with DCM (3 x 4 mL) followed by N-capping solution (4 mL) to block the unreacted resin. The mixture was allowed to shake for 1 hour. [N-Capping solution preparation: DCM:MeOH:DIPEA-17:2:1 respectively]. Then after removing the N-capping solution, the resin was soaked in NMP solvent (3 mL) and was allowed to shake for 15 min. After removing the solvent, the syringe was filled with a 25% piperidine solution in NMP (2 mL) and was allowed to shake for 10 min. After removing the piperidine solution, the resin was washed thrice with NMP solvent. Then the syringe was filled with the pre-activated Fmoc amino acid in NMP (3 mL) and was allowed to shake for 1 hour. [Pre-activation of Fmoc-AA: To a solution of Fmoc-AA (1 mmol) in DCM (2 mL) was added DIEA (1.2 mmol) followed by HATU coupling reagent (1 mmol) in NMP (1 mL), and the solution was allowed to sit for 10 min. The pre-activation was completed similarly for all subsequent Fmoc-amino acids] Washings between deprotection, coupling, and subsequent deprotection steps were carried out with NMP (3 × 1 min) using 3 mL of solvent per 0.2 mmol of resin for each wash. After completion of the amino acid backbone sequence, the resin was washed with DCM (2 x 3 mL). The syringe was then filled with cleavage cocktail (2 mL) and was allowed to shake for 1 hour. [Cleavage cocktail preparation: TFA:H_2O:TIPS-380:1:1respectively.] Then the cleavage cocktail was collected in a small glass vial and evaporated. The crude compound was purified by preparative-HPLC [Solvent system: water:acetonitrile (90:10); Flow rate: 40 mL/min; UV detector wave length: 254 nm] to afford pure final product.

Analytical LCMS data for final compounds were acquired using an Agilent 6110 Series system with the UV detector set to 220 and 254 nm. Samples were injected (<10 mL) onto an Agilent ZORBAX Eclipse Plus 4.6 x 50 mm, 1.8 µm, C18 column at room temperature. Mobile phases A (H_2O + 0.1% Acetic acid) and B (ACN + 1% H_2O + 0.1% Acetic acid) were used with a linear gradient from 10% to 100% B in 5.0 min, followed by a flush at 100% B for another 2 minutes with a flow rate of 1.0 mL/min.

UNC3866-PEG4-acid (UNC7126)

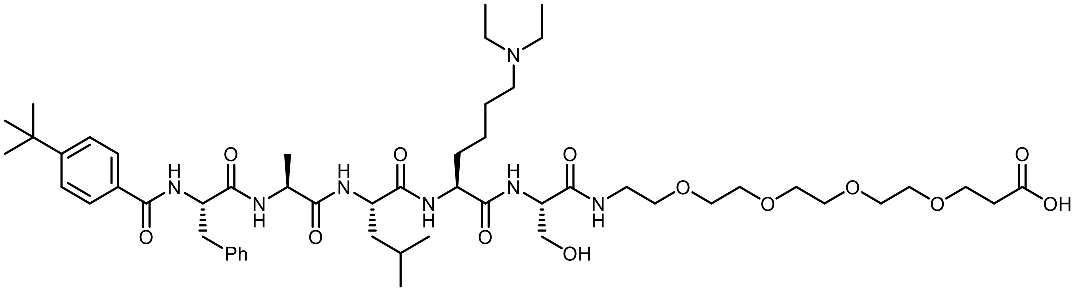

UNC3866-PEG4-acid (UNC7126): Chemical Formula: $C_{53}H_{85}N_7O_{13}$; Molecular Weight: 1028.30; LCMS (M+1): 1029.5; Yield: 22%

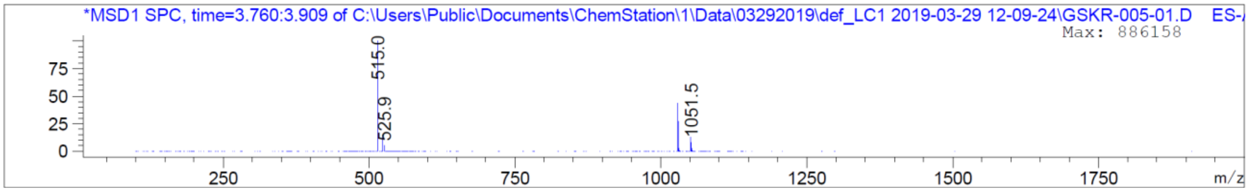

UNC4219-PEG4-acid (UNC7131)

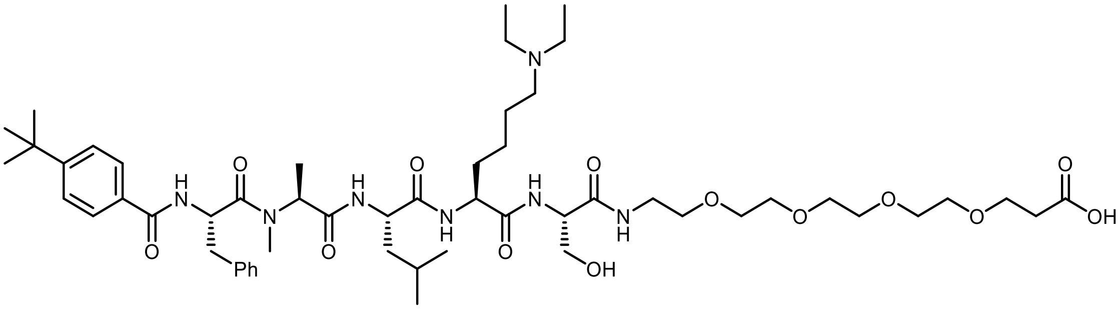

UNC4219-PEG4-acid (UNC7131): Chemical Formula: $C_{54}H_{87}N_7O_{13}$; Molecular Weight: 1042.33; LCMS: (M+1): 1043.5; Yield: 21%

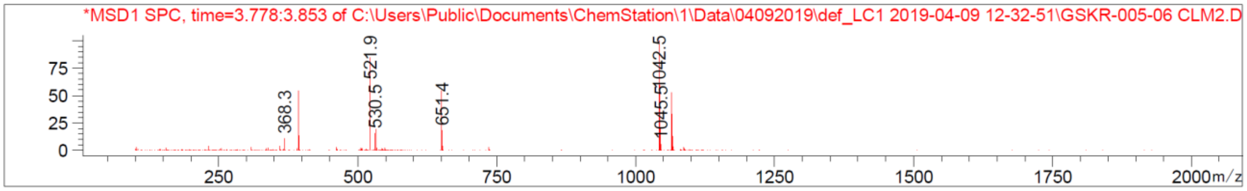

Coupling of control compounds to DNA

To 6000 pmol DNA-scaffold-IP003-Atag in coupling reaction buffer (50 mM MOPS, pH 8.0, 0.5 M NaCl, 96 μL) was added a mixture of UNC7126 or UNC7131 (60 mM, 60 μL), EDC (300 mM, 12 μL), HOAt (60 mM, 12 μL) and DIPEA (300 mM, 24 μL) in DMSO, previously activated for 15 minutes at room temperature. The reactions were agitated at room temperature for 18 hours. The reactions were then treated with a second addition of freshly activated acid in DMSO (same activation mixture as above) and agitated for 6 hours at room temperature. The reactions were quenched by addition of NH_4OAc (500 mM, 36 μL) at room temperature for 30 minutes and the DNA was isolated by ethanol precipitation. Samples were resuspended into 500 μL of MB grade water and desalted using NAP-5 columns to remove excess reagents and salts from the sample. The desalted sample was dried again using a speed-vac to concentrate. The sample was resuspended into 35 μL of molecular biology grade water.

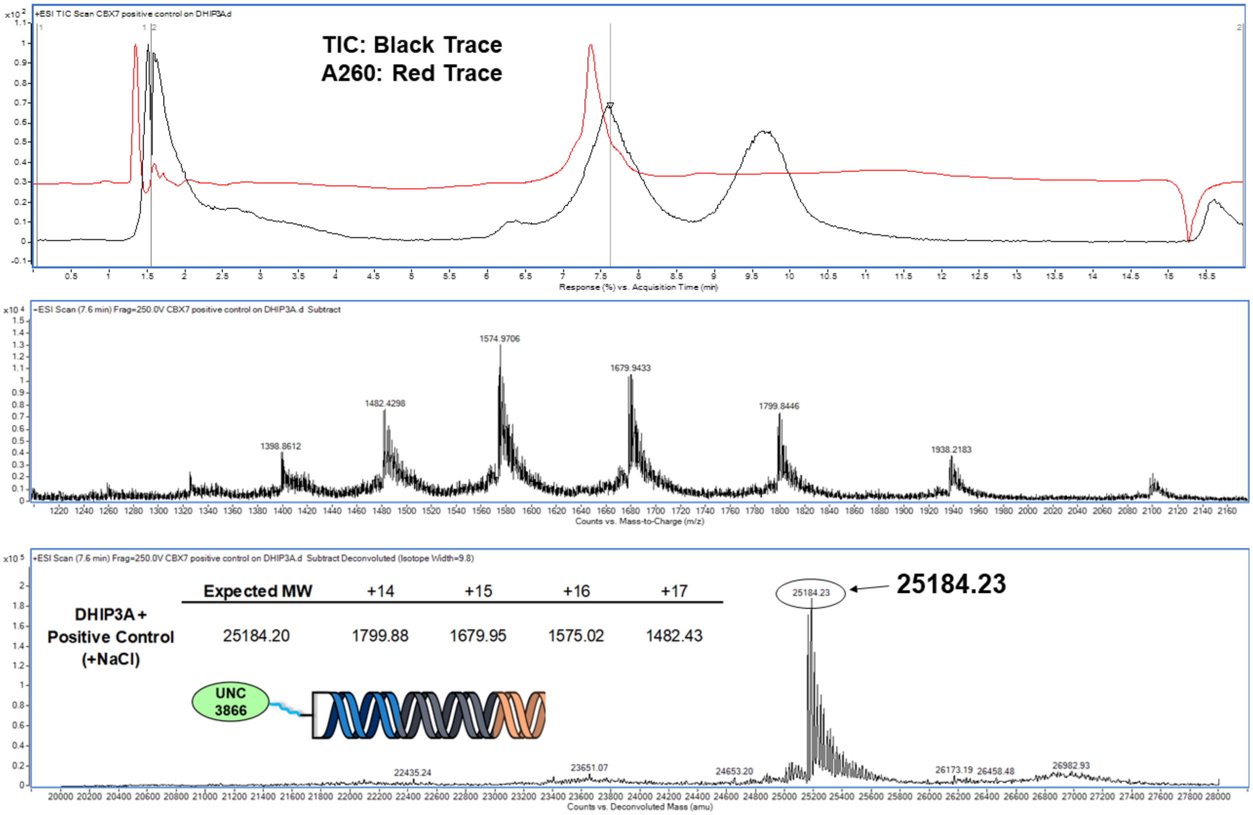

Figure S8. LC-MS trace and ionization pattern of the CBX7 positive control coupled to DNA-scaffold-IP003-Atag with deconvoluted mass.

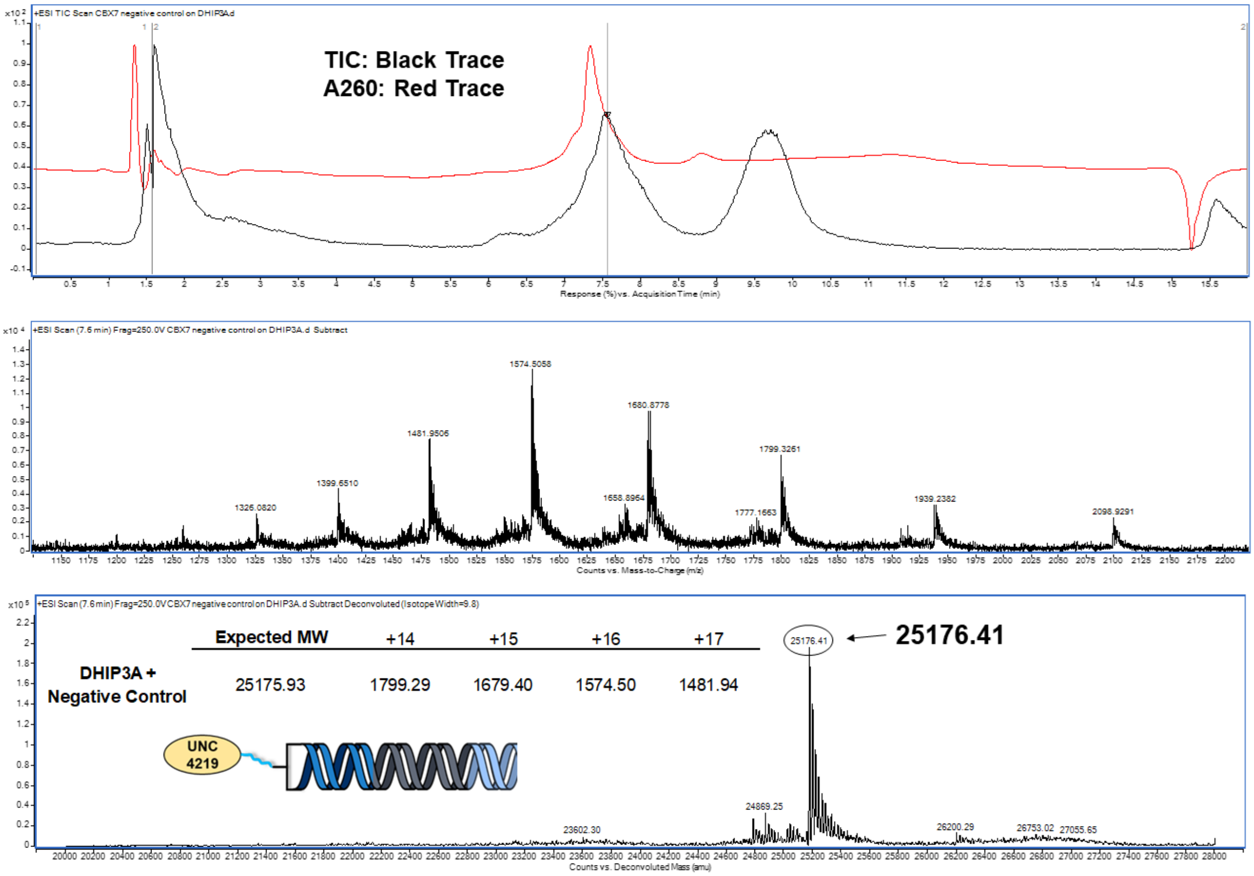

Figure S9. LC-MS trace and ionization pattern of the CBX7 negative control coupled to DNA-scaffold-IP003-Atag with deconvoluted mass.

Ligation of tags for control compounds

The DNA barcodes for the CBX7 positive and negative control compounds were ligated using the same reagent conditions as the library production. The DNA to which UNC7126 and UNC7131 were DNA-scaffold-IP003-A36 and DNA-scaffold-IP003-A37 respectively. The positive control compound barcode incorporated B3 and the negative control compound incorporated B4. The positive control barcode incorporated C46 and the negative control compound incorporated C47. ClosingPrimer003 was ligated to the ends of the barcodes and filled-in using Klenow polymerization.

qPCR experiments for CBX7 control compounds

A typical qPCR experiment of 25 µL contained 1 nM of barcoded control, 500 nM of each PCR primer, and 1X SsoAdvanced™ Universal SYBR® Green Supermix diluted with molecular biology grade water. The thermocycler was set-up to perform the following: 5 min at 95 °C, then 35 cycles of 30 s at 92 °C; 15 s at 55 °C; 15 s at 72 °C, followed by 10 min at 72 °C. qPCR experiments of samples from selections were completed in duplicate.
